# Pharmacological Targeting of GLUT1 to Control Autoreactive T Cell Responses

**DOI:** 10.3390/ijms20194962

**Published:** 2019-10-08

**Authors:** Carla Di Dedda, Debora Vignali, Lorenzo Piemonti, Paolo Monti

**Affiliations:** San Raffaele Diabetes Research Institute, IRCCS Ospedale San Raffaele, 20133 Milan, Italy; didedda.carla@hsr.it (C.D.D.); vignali.debora@hsr.it (D.V.); piemonti.lorenzo@hsr.it (L.P.)

**Keywords:** type 1 diabetes, T cells, autoimmunity, Glut1

## Abstract

An increasing body of evidence indicates that bio-energetic metabolism of T cells can be manipulated to control T cell responses. This potentially finds a field of application in the control of the T cell responses in autoimmune diseases, including in type 1 diabetes (T1D). Of the possible metabolic targets, Glut1 gained considerable interest because of its pivotal role in glucose uptake to fuel glycolysis in activated T cells, and the recent development of a novel class of small molecules that act as selective inhibitor of Glut1. We believe we can foresee a possible application of pharmacological Glut1 blockade approach to control autoreactive T cells that destroy insulin producing beta cells. However, Glut1 is expressed in a broad range of cells in the body and off-target and side effect are possible complications. Moreover, the duration of the treatment and the age of patients are critical aspects that need to be addressed to reduce toxicity. In this paper, we will review recent literature to determine whether it is possible to design a pharmacological Glut1 blocking strategy and how to apply this to autoimmunity in T1D.

## 1. Introduction

Type 1 diabetes (T1D) is an autoimmune disease caused by the selective destruction of insulin producing beta cells [1]. Both CD4+ and CD8+ T cells were implicated in beta cell destruction. Earlier studies detected CD4+ T cells against insulin and GAD65 in the pancreatic lymph nodes or islets obtained from patients with T1D [2]. More recently, CD8+ T cells reactive to multiple beta-cell antigens were detected in the islet infiltrate in the pancreas of patients, with an important role in the process of beta-cell killing [3]. Therefore, both CD4+ and CD8+ T cells specific for beta cell antigens are considered a major target for immune-based therapies aiming to control the autoimmune process [4,5,6,7]. The field of immunotherapy for T1D did not develop significantly until the 1980s, when studies using cyclosporine have shown that it is possible to control the autoimmune reaction. However, the toxicity of the treatment was considered a barrier to the application of cyclosporine treatment to the vast majority of cases of T1D. Since then, many other trials have been performed using immune-suppressive or immune-modulating drugs, and antigen specific approaches trying to balance efficacy with manageable toxicity, but with limited results [8]. Only recently, a phase 2 trial reported that treatment with teplizumab (an Fc receptor–nonbinding anti-CD3 monoclonal antibody) delayed progression to clinical T1D in high-risk subjects [9] indicating that targeting T cells is still the main road to achieve control of autoimmunity. The diabetes research community and funding agencies are highly committed to this task, and organized infrastructure an example of which is the TrialNet (http://www.diabetestrialnet.org). The aim is to develop immune-therapies based on immune-modulating drugs, antigen specific approach, and combination of both that prevent T1D or preserve metabolic function remaining at diagnosis. A major task is also the identification of novel cellular and molecular pathways that can be targeted to improve the control of autoimmunity. In this field area, an emerging field of research is the bio-energetic metabolism of T cells.

In recent years, there was an ever-growing body of data indicating that the T cell response is tightly linked to bio-energetic metabolism, with important implication for translation into immunotherapies [10,11]. Resting T cells rely on oxidative phosphorylation for basal energy production, whereas activated T cells predominantly use the anabolic pathway of glycolysis to support cell growth and proliferation. The metabolic differences of resting and activated T cells suggest the possibility to selectively target activated T cells, sparing quiescent T cells. Moreover, the extraordinary metabolic needs of activated T cells render them vulnerable to apoptosis and exhaustion, if bioenergetics needs are not fulfilled. In activated T cells during the glycolytic phase, Glut1 is the main glucose transporter for glucose uptake to fuel the glycolytic pathway [12]. Importantly, as Glut1 is considered an important target molecule in cancer cells, a growing number of small molecules that act as selective inhibitors of Glut1 are becoming available.

In this paper, we will review recent literature on the expression, dynamic, and relevance of Glut1 in T cells to evaluate the potential therapeutic value of pharmacological Glut1 blockade for T1D. Ideally an immunotherapy has to be non-toxic, limited in time, selective for target T cells, and impart permanent or long-term effect. As the majority of cells in the body express Glut1, it is important to evaluate potential off-target and side effects that may limit the translational potential of this strategy. All these aspects will be considered to determine whether, with the current knowledge, we are in the position to envisage an immunotherapy for T1D based on pharmacological Glut1 blockade.

## 2. Brief Overview of T Cell Metabolism

**Figure 1:**
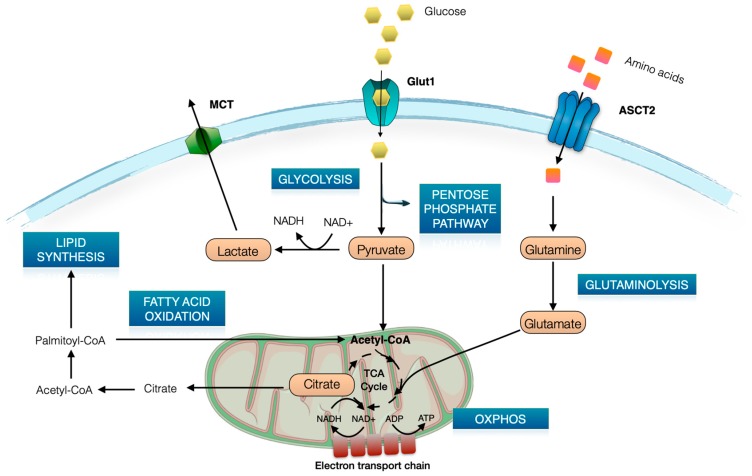
Schematic summary of the metabolic network. ATP can be generated from glucose though two integrated pathways. Glucose enters the cell via Glut1 and undergoes to enzymatic breakdown to pyruvate in the glycolysis pathway in the cytoplasm. The tricarboxylic acid (TCA) cycle encompasses the second pathway, where pyruvate is converted to acetyl-CoA in the mitochondria to fuel oxidative phosphorylation (OXPHOS). Anaerobic glucose catabolism transforms pyruvate into lactate that is transported out of the cell. Other substrates can also be metabolized in the TCA cycle, such as fatty acids via β-oxidation and glutamine via glutaminolysis.

Cells regulate their metabolic activity to generate energy and biosynthesis intermediates for survival and proliferation [13] (Figure 1). Glycolysis and oxidative phosphorylation (OXPHOS) are the two biochemical processes in the cell to generate chemical energy in the form of ATP. Glycolysis begins with the uptake of glucose from the extracellular milieu for conversion into pyruvate through a series of intermediate metabolites that can alternatively enter the pentose phosphate pathway, thus contributing to biosynthetic processes for cell growth [14]. Pyruvate can be transported into the mitochondria and converted into acetyl-CoA, which enters the tricarboxylic acid (TCA) cycle. Alternatively, pyruvate can be converted into lactate in the cytoplasm, which is ultimately excreted from the cell. Glycolysis is also involved in the reduction of NAD+ to NADH, thus contributing to the redox balance of the cell.

In quiescent state, T cells produce ATP through mitochondrial OXPHOS. This process requires that substrates such as lipids and amino acids are transformed to generate metabolic intermediates that are subsequently oxidized in the TCA cycle [15]. Lipids enter the fatty acid oxidation (FAO) process and are converted into intermediates that enter the TCA cycle. Moreover, amino acids such as glutamine can be metabolized to produce intermediates for the TCA cycle. Glutaminase is the enzyme that converts glutamine into glutamate that is transported in mitochondria through the mitochondrial glutamate transporter and is used in the TCA cycle. TCA cycle oxidize substrates to produce the coenzymes NADH and FADH2 that gives electron to the electron transport chain to sustain OXPHOS. The TCA cycle also produces citrate as intermediate for biosynthetic pathways such as fatty acids synthesis [16]. ATP production in the glycolytic pathways is 10 times less efficient than OXPHOS.

The T-cell decision to use glycolysis or OXPHOS is dictated by the needs for activation, differentiation, and proliferation to find the optimal balance for ATP production, biosynthetic substrates, and redox conditions. Quiescent T cells have basal bio-energetic needs that are supported by oxidative phosphorylation, fueled by fatty acid oxidation. Upon activation, T cell metabolism is reprogrammed to glycolysis that better fits the increased energetic demand and the needs for biosynthetic intermediates necessary for cell growth and division [17]. Central to the metabolic switch to glycolysis is the surface expression of the facilitative glucose transporter Glut1.

## 3. Glut1 Expression, Usage, and Blockade in T Cells

**Figure 2:**
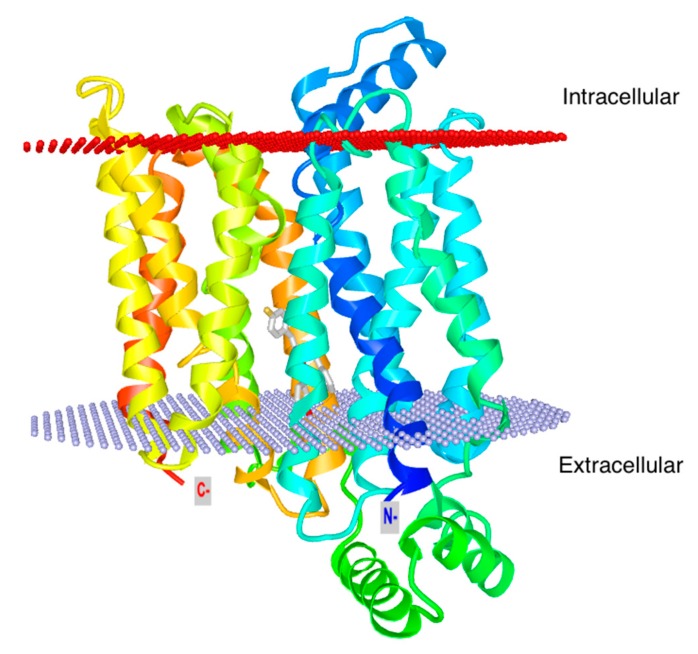
Glut1 structure. Ribbon model of GLUT1 in the ligand-bound inward facing conformation (PDB: 4PYP; https://www.rcsb.org/structure/4PYP). The N terminus is colored in blue and the C terminus in red. The corresponding transmembrane segments in the four 3-helix repeats are colored the same. The position of glucose bound in the inward facing state is depicted in gray sticks. The structure figure is customized with iCn3D.

Glucose transport across the cell membrane is a facilitated transport mediated by a family of at least 14 trans-membrane carrier proteins named glucose transporters [18]. These transporters modulate the thermodynamically downhill movement of glucose according to the concentration gradient, and are therefore bi-directional. Glut1 is present in nearly all mammalian cells with a Michaelis–Menten constant (Km) about 1mM. This is less than the normal blood glucose level (5.5mM), resulting in the continuous transport of glucose inside cells at an essentially constant rate. The crystal structure of Glut1, as well as predicted conformations have been recently reported by Deng [19] (Figure 2). Glut1 is encoded by the *Slc2a1* gene, and consists of a sugar-binding pocket facing the outer cell in the outward open conformation. Binding of glucose causes a conformational change so that Glut1 opens into the cytoplasm and releases glucose inside the cell.

**Figure 3:**
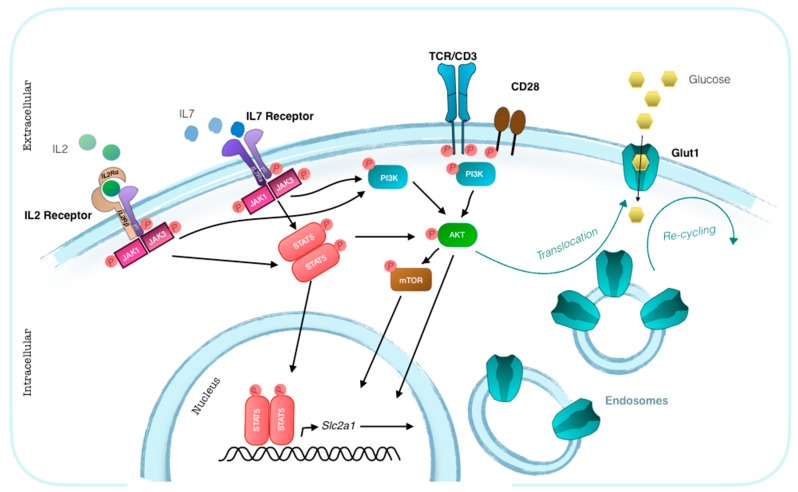
Glut1 expression and trafficking in T cells. The T cell surface expression of Glut 1 is regulated by extrinsic signals. The transcription of the Slc2a1 gene is induced by engagement of TCR and CD28 co-stimulation or by cytokine signaling through phosphorylated STAT5. The translocation of the intracellular pool of Glut1 to the cell surface is mainly regulated by Akt. Akt activation is the result of TCR and CD28 engagement or can be activated by phosphorylated STAT5 through the IL-2 or IL-7 signaling pathways.

Of the fourteen members of glucose transporter family, T cells express Glut1, 3, 6 and 8 [12]. Glut1 is expressed at low levels on the surface of resting T cells and is up-regulated upon T cell activation. Similar to the insulin-responsive glucose transporter Glut4, Glut1 cell surface localization is controlled by extrinsic signals [20] (Figure 3). In addition to TCR signaling, co-stimulation via CD28 engagement induces the expression and surface localization of Glut1 in T cells through the activation of the phosphoinositol-3 kinase (PI(3)K)-Akt pathway [21]. T cells have a cytoplasmic pool of Glut1 whose translocation to the cell surface is responsible for increased glucose uptake peaking at 48/72 h after activation [22]. This kinetic indicates that Glut1 translocation to the cell membrane is predominantly driven by the autocrine IL-2 production, and up-regulation of CD25 rather than directly from TCR engagement. Translocation of Glut1 to the cell membrane can indeed be induced also by stimulating resting T cells with the homeostatic cytokine IL-7, in the absence of antigenic or co-stimulatory signals [23]. In the absence on an immune response, IL-7 maintains the basal levels of Glut1 expression necessary for T cell survival. Glut1 trafficking is promoted by canonical common γc signaling [24]. The crosslink of IL-7 with the extracellular domains of IL-7Rα and γc results in the interaction of the intracellular domains of the two chains. Tyrosine kinases Janus kinase 1 (JAK1) and JAK3, which are linked to the γc intracellular domain phosphorylate each other and increase their kinase activity to phosphorylate the intracellular domain of the IL-7Rα. This allows the signaling molecule signal transducer and activator of transcription 5 (STAT5) to bind the IL-7Rα complex. Phosphorylation of STAT5 allows its dimerization and subsequent translocation to the nucleus where it acts as a key promoter of gene transcription. STAT5 mediated activation of Akt has a central role in regulating Glut1 trafficking, leading to the increased surface expression of Glut1 [23].

Despite the expression of four different Gluts on the T cell surface, conditional deletion of the Slc2a1 gene showed that Glut1 has a fundamental and non-redundant role in activated effector T cells expansion [12]. The impaired proliferation of T cells lacking Glut1 leads to defective generation of Th1, Th2, and Th17 cells both in vitro and in vivo. Conversely, resting T cells express Glut1 at lower levels than activated T cells, and they remained unaffected by genetic knock down. Similarly, lack of Glut1 did not affect the presence and survival of CD4+CD25+ regulatory T cells. Glut1 expression is required not only for differentiation of T cells with full effector function but also for the generation of long-lived memory clones. Naïve T cell precursors stimulated with IL-7 generate long-lived memory stem T cells (Tscm) [25]. If Glut1 is inhibited, the capacity of proliferating naïve precursors to generate Tscm is severely impaired. Of importance, a similar impairment can be obtained also by blocking the mitochondrial pyruvate carrier (MPC) with the small molecule UK5099. This indicates that, unlike effector T cells, Tscm converts glucose into pyruvate that is subsequently transported into the mitochondria for oxidative metabolism. Corroborating this, Tscm do not produce significant amount of lactate despite high internalization of glucose. These data indicate that Glut1 blockade could be an effective strategy to prevent both the generation of activated effector T cells, but also the differentiation of long-lived Tscm.

Informative data with respect to the in vivo effects glucose deprivation in T cells have been generated in cancer models. The intense glycolytic activity of cancer cells can severely reduce the extracellular glucose concentration in the tumor microenvironment. Using a sarcoma model in which the glucose concentration in the tumor microenvironment was as low as 2mM, tumor-infiltrating T lymphocytes (TILs) underwent to a loss of function, due to altered metabolism resulting from tumor-imposed glucose restriction [26]. T cells from patients with acute or chronic B cell leukemia display reduced Akt/mammalian target of rapamycin complex 1 (mTORC1) signaling and a consequent reduced expression of Glut1 [27]. This metabolic impairment was associated to an exhausted phenotype, with increased expression of inhibitory receptors, such as programmed cell death protein 1 (PD-1), lymphocyte-activation gene 3 (LAG3), T-cell immunoglobulin, and mucin domain 3 (TIM3). T cell exhaustion is a state of T cell dysfunction in which sustained expression of inhibitory receptors lead to an impairment of proliferation and effector functions. Both leukemic and stromal cells in the tumor microenvironment express PD-L1 and PD-1 engagement of T cells was shown to suppress glycolysis and increases the rate of fatty acid beta-oxidation (FAO) [28]. The rescue of T cell metabolism by genetically increasing expression of Glut1 partially restored T cell function [27]. These data suggest that limiting the availability of glucose to T cells result in an exhausted phenotype and function, providing a rationale to perform a pharmacological Glut1 blockade to control the T cell response.

## 4. Metabolic Modulators in the Treatment of Autoimmunity

Inhibition of glycolysis as immunotherapy approach to autoimmunity has been tested in animal models of autoimmune diseases, mostly using 2-Deoxy-D-glucose (2DG) in combination with other drugs. 2DG is a glucose analog that is uptaken through glucose transporters and is phosphorylated by hexokinase to 2DG-6-phosphate inside the cell. 2DG-6-phosphate cannot be further metabolized via glycolysis, but accumulates and noncompetitively inhibits hexokinase and competitively inhibits phosphogluco-isomerase [29]. 2DG preferentially target effector T cells that upregulated Glut1 expression and glycolytic activity upon activation [30]. Gene array analysis of CD8+ T cells treated with 2-DG showed inhibition of the expression of genes for key cytokines, cell cycle molecules, and cytotoxic granule proteins [31]. Consistent with these results, IFN-γ and GM-CSF secretion, cell cycle progression, upregulation of cyclin D2 protein, cytolytic activity, and upregulation of granzyme B were significantly affected by suppression of glycolysis. 2DG treatment preferentially reduce the generation and function of highly glycolytic effector T cells and can result in enhanced generation of memory T cells [32].

Systemic lupus erythematosus (SLE) is characterized by the presence of CD4+ T cells in a state chronic activation with active glycolysis and mitochondrial metabolism. Treatment of B6.Sle1.Sle2.Sle3 (TC) mice with a combination of metformin and 2DG led to normalization of T cell metabolism and reversed disease biomarkers [33]. A study conducted in the K/BxN mouse model of spontaneous rheumatoid arthritis showed that preventive inhibition of T cell metabolism with 2DG significantly reduced joint inflammation and the activation of both adaptive and innate immune cells, as well as the production of pathogenic autoantibodies [34]. A study conducted in a mouse model of experimental autoimmune encephalomyelitis (EAE) showed that metformin treatment attenuates clinical signs of EAE and associates with reduced pathogenic Th17 cell and increased Foxp3+ regulatory T cells secreting TGF-β and IL-10 [35]. The mechanism of action of metformin included the suppression of the mTOR pathway and its downstream target hypoxia-inducible factor transcription factor 1α (HIF-1α).

Treatment with 2DG has been tested in the non-obese diabetic (NOD) mouse model, which develop spontaneous autoimmune diabetes. In this model, diabetogenic CD8+ T cells specific for a peptide from the diabetes antigen IGRP (NRPV7- reactive) have features of activated memory T cells, including an increased glycolytic rate and oxidative phosphorylation. Treatment of these mice with 2DG resulted in a reduced frequency of activated T cell, reduced immune infiltration within the islets, and in improved β-cell granularity [36]. Although these findings are still preliminary, preclinical models of autoimmune conditions revealed the presence of an enhanced metabolic activity of autoreactive T cells. Even though the activation of specific metabolic pathways in T cells vary in different models, metabolic inhibition was often effective in the control of the immune reaction. This indicates that targeting metabolism and especially glycolysis is a promising therapeutic avenue for autoimmune disorders.

## 5. Novel drugs targeting Glut1

A number of natural or synthetic Glut inhibitors have been discovered over the years and a comprehensive review can be found elsewhere [37]. In this section, we provide a summary of the latest progress in the field, focusing especially on new generation molecules that specifically target Glut1. Three small molecules gained interest in recent years (Table 1).

STF-31 selectively inhibits the glucose transporter Glut1 and selectively impairs cell growth of kidney and other types of cancer cells that lack the von Hippel-Lindau (VHL) tumor suppressor protein [38]. Inactivation of VHL increases the expression of the hypoxia-inducible factor transcription factor HIF, which in turn stimulates the transcription of genes involved in glucose metabolism, including the Glut1 gene. VHL-deficient cancer cells depend on Glut1 and aerobic glycolysis for ATP production. STF-31 binds directly to the Glut1 transporter, blocking glucose uptake, resulting in necrosis in VHL-deficient cancer cells. Normal kidney cells are not strictly dependent on glycolysis, use Glut2 for glucose uptake, and are therefore insensitive to STF-31 toxicity. When used in a mouse model, STF-31 efficiently blocked tumor growth without significant toxic effect on other organs. STF-31 was shown to efficiently inhibit Glut1 dependent glucose uptake and to suppress glycolysis in human T cells overexpressing Glut1 [39]. Moreover, STF-31 was shown to impair glucose responsiveness and insulin secretion in human beta cells expressing Glut1 [40].

WZB117 is a small molecule that inhibits Glut1-mediated glucose transport by binding reversibly at the exofacial sugar-binding site of Glut1 [41]. WZB117 was shown to induce cell death in lung and breast cancer cells without affecting normal cells [42]. As for STF-31, in vivo treatment of animal models with WZB117 affected tumors without causing significant adverse events in treated animals. We used WZB117 to inhibit Glut1 mediated glucose transport in T cells [25]. WZB117 reduces T cell proliferation by 90% at a concentration of 3µM and differentiation of naïve T cells into memory T cells. Even though both STF-31 and WZB117 have proven effective both in vitro and in pre-clinical models, their Glut1 blocking activity in the µM range and the controversial selectivity for Glut1 [41,43] represent significant limitations for their translation into the clinic.

So far, the most promising molecule for translation into the clinical setting is BAY-876 [44]. It is characterized by an IC50 of 2 nM, and it is highly selective for Glut1 with a selectivity factor of >100 against Glut2, Glut3, and Glut4. Importantly, it is orally bioavailable, and showed a good metabolic stability in vitro. As for the two other compounds, it was successfully tested in preclinical models, showing a potent anti-cancer activity [45]. At the moment, no data are available with respect to the effect of BAY-876 on cells of the immune system.

## 6. Potential off-Target and Side Effects of Pharmacological Glut1 Blockade

The majority of the cells in the body express Glut1, but the effect of pharmacological Glut1 blockade largely depends on the co-expression of other Gluts, and on the metabolic activity of the cell, which determine the need for glucose. Two cell types that are strictly Glut1 dependent are erythrocytes and the endothelium of the blood brain barrier (BBB). Of all cell lineages, the human erythrocyte expresses the highest level of the Glut1 with a surface density of approximately 200.000 Glut1 molecules per cell [46]. In addition to glucose, Glut1 also transports L-dehydroascorbic acid (DHA, vitamin C), and in human erythrocytes there is a preferential uptake of DHA instead of glucose [46]. Glut1 expression in erythrocytes is a specific trait of vitamin C–deficient mammalian species, comprising only higher primates, guinea pigs, and fruit bats. In DHA synthetizing mammals, Glut1 expression is down-regulated after the neonatal period. These data suggest that the high expression of Glut1 in human erythrocytes in not related to a specific need for glucose when cells have a modest metabolic activity. Accordingly, treatment of human erythrocytes with the Glut1 inhibitor STF-31 significantly impaired glucose uptake but did not cause hemolysis or other effects [38].

The capillary endothelium of the brain, which makes up the BBB express Glut1, with a fundamental role in the transport of glucose from the blood to the central nervous system (CNS). The Glut1 deficiency syndrome (Glut1-DS) is the most relevant clinical setting showing the non-redundant role of Glut1 function in the BBB. The Glut1-DS is a rare genetic disease characterized by de novo or inherited mutations (approximately 100 identified) of the SLC2A1 gene. Mutations affect assembly, three-dimensional folding, trafficking to the cell membrane, or activation of the encoded protein Glut1 [47,48]. The extent of Glut1 dysfunction can be measured as in vitro uptake of glucose in erythrocytes showing that glucose transport is reduced by 30% to 70% as compared to healthy subjects [49]. The disease has typical neurological symptoms including ataxia, lethargy, total body paralysis, movement disorders, and epilepsy. Neurological symptoms similar to those reported in patients with the Glut1-DS have to be considered as potential side effects when patients undergo to pharmacological Glut1 blockade. However, in the Glut1-DS most of the neurological symptoms are developed during childhood and in the adolescence, and later in adulthood most of the symptoms stabilize or even attenuate [50]. The impact of genetic Glut1 deficiency on neurological damage appear to be related to the relative glucose shortage in the developing CNS, while once completely developed the impact of reduced glucose availability is limited. In terms of translation of a pharmacological blockade strategy in patients with T1D, the effects on the CNS have to be considered as the most relevant off-target effect, with important complications. To avoid neurological side effects pharmacological Glut1 blockade should be restricted to adolescent or adult patients and for a limited time period. Supporting this, clinical trials using 2DG in adult patients for several weeks, reported only mild and reversible neurological side effects, mainly dizziness [51].

A diabetes-specific side effect of pharmacological Glut blockade that has to be considered is the potential inhibition of insulin production by beta cells. Human, but not rat and mouse insulin producing beta cells express Glut1 [52]. Glut1 is used as glucose sensor for optimal insulin secretion and inhibition of Glut1 with STF31 resulted in impaired insulin secretion [40]. Even though the effect of Glut1 inhibitors is reversible this suggests that pharmacological Glut1 blockade can only apply for a limited time period in order to restore proper insulin production. Moreover, nicotinamide phosphoribosyltransferase (Nampt), a rate-limiting enzyme in the mammalian NAD+ biosynthesis, has been suggested as an alternative target for STF-31 [43]. While staining for Nampt was found both in the exocrine and endocrine tissue of fetal pancreas, in adulthood, Nampt expression was localized predominantly in beta cells [53]. These data suggest that beta cells are the main target for Glut1 blockade in human islets. Other potential target cells within the islet of Langherhans are alpha cells that were shown to express Glut1 [54], but so far the effect of Glut1 blocking compounds on alpha cells has not been investigated.

Potential off-target and side effects of a pharmacological Glut1 blockade have not been addressed in humans, and animal models not always resemble humans for Glut1 expression and usage. An ideal model to study immune and endocrine effects of a reduced glucose transport through Glut1 is the Glut1-DS. This model has been extensively characterized for the neurological symptoms but so far no efforts have been made to address potential impairments in other cells and organs.

## 7. Pharmacological Glut1 Blockade to Target Autoreactive T Cells in Type 1 Diabetes

The rationale of a Glut1 blocking strategy to control autoimmunity in patients with T1D is based on two key observations. First, the extreme need for glucose of activated T cells make them vulnerable to glucose shortage more than other cells. Second, the exhausted phenotype and function induced by glucose deprivation is a long-term effect that persist after the Glut1 inhibitor is removed. As only activated effector T cells display a significant surface Glut1 expression and have a glycolytic metabolism, it is of fundamental importance to determine time windows for intervention with Glut1 blocking drugs. CD4+ T cells specific for GAD65 and proinsulin are already present at birth [55], but they develop a memory phenotype and telomere shortening (marker of in vivo expansion) in patients positive for autoantibodies before the onset of the disease [56]. The time of seroconversion could be valuable to determine the time-window for conversion of quiescent naïve T cells into proliferating effectors and later to memory T cells.

Considering the potential impact of off-target and side effects, the Glut1 blocking strategy should be designed to be a short-term treatment for adult patients to induce a long-term effect on activated T cells without affecting, or reversibly affecting other cells and organs in the body. Moreover, Glut1 blockade would better work if all target T cells are in an activated and glycolytic phase in a defined time window, in order to design a treatment that is limited in time. T1D is a chronic autoimmune disease in which a slow or perhaps relapsing/remitting activation of autoreactive T cells lead to the destruction of the beta cell mass in a long time period. The time from the appearance of signs of autoimmunity (autoantibodies) to the diagnosis of the disease, when 70% to 80% of the beta cell mass has been destroyed is typically measured in years. In addition, several patients are very young increasing the possibility of neurological involvement as a consequence of Glut1 blockade.

**Figure 4:**
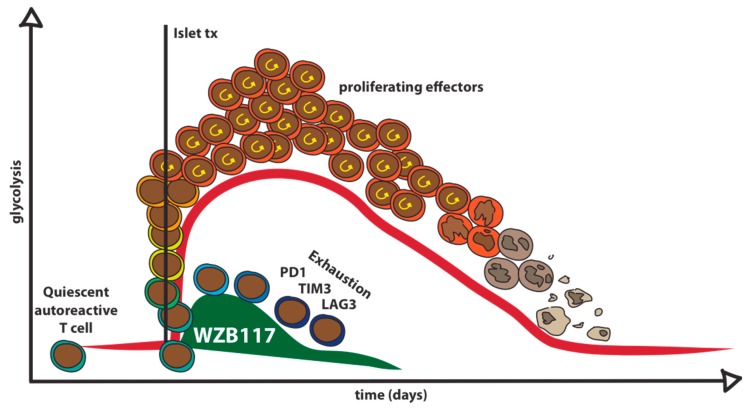
Graphical model of Glut1 blockade approach in patients with T1D undergoing to islet transplantation. Resting memory clones are activated by islet transplantation and rapidly increase glucose uptake via Glut1 to fuel glycolysis necessary for expansion, proliferation, and effector functions. Treatment with the Glut1 inhibitor WZB117 prevents metabolic re-programming to glycolysis and failure to fulfill bio-energetic needs drive T cells in a state of anergy and exhaustion.

In this context, a much more feasible clinical setting is islet or pancreas transplantation. For the most severe cases of T1D, allogenic islet [57] or pancreas [58] transplantation are therapeutic options to replace the beta cell mass. Islet or pancreas transplantation in patients with T1D represents an immunological challenge in which activation of alloreactive T cells co-exist with the potential for re-activation of autoreactive memory T cells [59]. The relapse of autoimmunity is difficult to control with standard immunosuppression as assessed by measurements of autoantibodies and occasionally T cells following islet transplantation [60,61,62]. Importantly, patient candidates to islet or pancreas transplantation are adults, usually several years after the diagnosis. This is relevant to the activation state of autoreactive T cells that are in a resting mode before transplant and are activated after transplant by the exposure to beta cell associated antigens from the graft [59]. Therefore, the transplantation setting provides an optimal and well-defined time window for a Glut1 blockade strategy (Figure 4). Treatment with Glut1 inhibitor can be introduced as an induction therapy during the first two or three weeks post-transplant, and induce a durable exhaustion of autoreactive, and possibly alloreactive T cells. An important aspect that needs to be considered in further studies is the interaction between Glut1 inhibitors and standard immunosuppressive regimens. In islet transplantation maintenance therapy include rapamycin, FK506, and occasionally mycophenolate mofetil, all of which can potentially affect activation, proliferation, and metabolism of T cells [63]. The use of immunosuppressive drugs that cause a mild reduction of circulating T cells has been associated to homeostatic T cell proliferation driven by IL-7 [62,64]. Homeostatic proliferation is resistant to the inhibitory effect of standard immunosuppressive drugs [65] and was shown to drive the expansion of autoreactive memory T cell clones. Interestingly, T cells exposed to IL-7 up-regulate Glut1 and increase glucose uptake [23] rendering T cells more susceptible to Glut1 blockade [36].

In the transplantation context also the inhibition of insulin secretion induced by Glut1 blockade could exert a positive effect. Insulin production is considered a challenging process for beta cells, especially in stress conditions like ones generated by isolation procedure for islet transplantation, the inflammatory response early post-transplant, and the lack of vascularization in weeks post-transplant [66]. In these conditions, the reduction of glucose sensitivity, and reduced insulin secretion induced by Glut1 blockade in the first weeks post-transplant could potentially protect the graft from the massive loss of beta cells that occurs after islet transplantation.

Despite the model of transplanting islets or pancreas provides an ideal setting to test a pharmacological Glut1 blocking strategy to control the autoimmune response, the natural history of T1D represent the final aim of this strategy. A way to overcome most of the limitations and render T1D more similar to islet transplantation in terms of the dynamic of the immune response is to provide an antigenic challenge, to obtain a time defined and broad activation of autoreactive T cells. Clinical trials involving vaccination with beta cell associated antigens [67] or transplantation of islets as antigenic stimulation to promote tolerance in the presence of immune-modulating agents (https://clinicaltrials.gov/ct2/show/NCT02505893) could be an ideal clinical setting to transfer the Glut 1 blocking strategy in the natural history of T1D.

## 8. Feasibility and Potential Roadblocks

At the moment, the clinical translation of an immunotherapy based on Glut1 has to overcome several obstacles. Selective inhibitors of Glut1 have proven effective and safe when used in animal models, but data about pharmacokinetics and pharmacodynamics are limited, and none of the compounds have been tested in humans. So far, the best-characterized molecule is BAY876, for which in vivo pharmacokinetics data are available for rats and dogs. In addition to the high selectivity for Glut1 and effectiveness in the nanomolar range, both the good oral bioavailability and long terminal half-life of BAY876 make this small molecule attractive for further evaluation in a phase 1 clinical trial. Expected side effects of the approach in humans are most likely neurological symptoms, similar to those described in patients affected by the Glut1-DS. While these potential side effects can be quite severe, most of them can be efficiently controlled with ketogenic diet (KD), which represents the standard treatment for Glut1-DS patients [68]. Low-carb or KD regimens have also been evaluated in patients with T1D, showing benefits in the improvements of glycaemic variability, but limited long-term tolerability and patients compliance [68]. However, considering the length of an induction therapy in islet transplantation of three to four weeks, the introduction of a KD would be highly feasible. From the immunological stand-point, an important issue that needs to be addressed is the possibility that T cells acquire resistance to Glut1 blockade. Changes in the expression of other Gluts or metabolic switch to other substrates, such as lipids and amino acids, could cause a rapid acquired resistance to Glut1 inhibitors. Even though there are no evidences in the literature of such mechanisms, patients with the Glut1-Ds are clinically immune-competent and no increased risk of opportunistic infections of other defects of the immune system have been described.

## 9. Concluding Remarks

Metabolic manipulation is an attractive approach to control T cell responses in autoimmune diseases, including T1D. According to the recent literature on T cell metabolism, direct targeting of Glut1 with selective inhibitors is a possibility. However, careful consideration needs to be taken with respect to the potential off-target and side effects and the design of a treatment taking in consideration the age of patients and the characteristics of the T cell response. While in the islet transplantation model the time window for T cell activation is well defined, in the natural history of T1D the time of seroconversion might be informative with respect to the dynamic of T cell activation. The Glut1-DS is a clinical model that can provide very informative data about the effect of a reduced glucose uptake both in the immune and endocrine system, and possibly highlight mechanism of adaptation to a reduced glucose availability that can reflect mechanisms of acquired resistance to a pharmacological Glut1 blockade. Further studies are needed to explore these aspects and evaluate the feasibility of this approach.

## Figures and Tables

**Table 1:** List and principal characteristics of small molecules that act as Glut1 inhibitors.

Name	Structure	MW	IC50 (µ)	Characteristics	Human Cell Target (ref)
STF-31	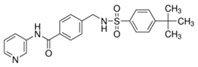	423.53	1	Low solubility	Renal cancer RCC4 [38] CD4+ T cells [39] Beta cells [40]
WZB-117	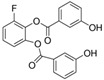	368.31	0.5	High solubility	Multiple cancer cell lines [42] Autoreactive CD8+ T cells [25]
BAY 876	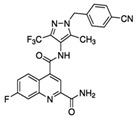	496.42	0.002	Highly selective Orally bioavailable	Colon cancer DLD1 [44]

## Abbreviations

T1D	Type 1 Diabetes
OXPHOS	Oxidative Phosphorylation
FAO	Fatty Acid Oxidation
TCA	Trycarboxylic Acid
Tscm	Stem Cell Memory T Cells
2DG	2-deoxy-D-glucose
SLE	Systemic Lupus Erythematosus
EAE	Experimental Autoimmune Encephalomyelitis
VHL	Von Hippel-Lindau
BBB	Blood Brain Barrier
CNS	Central Nervous System
GLUT1-DS	GLUT1 deficiency syndrome
